# Assessment of sagittal spinopelvic parameters in a Taiwanese population with spondylolysis by the EOS imaging system: a retrospective radiological analysis

**DOI:** 10.1186/s12891-021-04440-0

**Published:** 2021-06-18

**Authors:** Hao-Chun Chuang, Yu-Hsiang Tseng, Yueh Chen, Po-Hsin Chou, Wei-Lun Chang, Pei-Fang Su, Cheng-Li Lin

**Affiliations:** 1 Department of Orthopaedic Surgery, National Cheng Kung University Hospital, College of Medicine, National Cheng Kung University, No.138, Sheng-Li Road, 70428 Tainan, Taiwan; 2 Department of Plastic Surgery, Kaohsiung Veterans General Hospital, Kaohsiung, Taiwan; 3 Department of Orthopaedic Surgery, Sin Lau Christian Hospital, Tainan, Taiwan; 4 Department of Orthopedics and Traumatology, Taipei Veterans General Hospital, Taipei, Taiwan; 5 Division of Orthopaedics, Department of Surgery, National Cheng Kung University Hospital Dou Liou Branch, National Cheng Kung University, Yunlin, Taiwan; 6 Department of Statistics, National Cheng Kung University, Tainan, Taiwan; 7 Skeleton Materials and Bio-compatibility Core Lab, Research Center of Clinical Medicine, College of Medicine, National Cheng Kung University Hospital, National Cheng Kung University, Tainan, Taiwan; 8 Medical Device Innovation Center (MDIC), National Cheng Kung University, Tainan, Taiwan; 9 Musculoskeletal Research Center, Innovation Headquarter, National Cheng Kung University, Tainan, Taiwan

**Keywords:** Radiography, Spinopelvic parameter, Spinopelvic alignment, Spondylolysis, Spondylolisthesis

## Abstract

**Background:**

The impact of sagittal spinopelvic alignment on spondylolysis is well established in Caucasian populations. However, prior studies suggest that people from different ethnological backgrounds showed divergence, and a few studies that focused on Asian populations reported conflicting results. The aim of this study is to use the EOS imaging system to evaluate the spinopelvic parameters of spondylolysis patients, and their relationship with spondylolisthesis, disc degeneration, and age in a Taiwanese population.

**Methods:**

Radiographic sagittal spinopelvic parameters for 45 spondylolysis patients and 32 healthy people were evaluated, including pelvic incidence (PI), sacral slope (SS), pelvic tilt (PT), thoracic kyphosis (TK), and lumbar lordosis (LL). The spinopelvic parameters were compared between spondylolytic and control groups. These parameters were further compared between spondylolytic subjects with and without spondylolisthesis, with and without high-grade disc degeneration, and young (< 30 years old) and middle-aged.

**Results:**

The PI and LL of the spondylolytic group (52.6°±12.0° and 41.3°±15.2°) were significantly higher than those of the healthy control group (47.16°±7.95° and 28.22°±10.65°). Further analysis of the spondylolytic patients revealed that those with high-grade disc degeneration were more prone to spondylolisthesis (92.3 %) compared to those without (50 %; *p* = 0.001). The middle-aged group had significantly higher rates of spondylolisthesis (80 %) and high-grade disc degeneration (52.4 %) compared with those for the young group (45 and 16.7 %, respectively; *p* = 0.017 and 0.047, respectively). No statistically significant difference in the sagittal spinopelvic parameters was found when spondylolytic patients were divided according to the occurrence of spondylolisthesis or high-grade disc degeneration.

**Conclusions:**

In a Taiwanese population, PI and LL were significantly larger in spondylolytic patients. Disc degeneration and age were associated with the occurrence of spondylolisthesis. Ethnological differences should thus be taken into account when making clinical decisions regarding spondylolysis in a Taiwanese population.

## Introduction

Spondylolysis refers to a defect of the vertebral pars interarticularis. It can result in vertebral slippage relative to the adjacent vertebrae, a condition called spondylolisthesis. The pathogenesis of spondylolysis and spondylolisthesis was believed to involve sagittal spinopelvic alignment [1]. For example, an increase in pelvic incidence (PI) is correlated with lumbar lordosis in spondylolysis patients compared with healthy people [1–5] Also, the increase in PI is also correlated with an increasing slip grade in spondylolisthesis patients [4, 6–9]. Another common pathological change in spondylolysis patient is intervertebral disc (IVD) degeneration below the lytic level [10]. Some authors have concluded that the grade of slippage is positively correlated with spinopelvic parameters such as PI, sacral slope (SS), and lumbar lordosis (LL) in spondylolisthesis patients [11]. However, published studies focus mostly on Western populations. The few studies addressing the Eastern populations reported conflicting results [11–14].

Published studies used computed radiography (CR) or digital radiography (DR) to evaluate the spinopelvic parameters, spondylolisthesis, and spondylolysis. CR and DR systems project information on the image plane through a conic perspective. This causes distortion from the center to the edges of the radiograph, increasing errors in scale for structures far from the central region [15]. The EOS imaging system, a slot-scanning radiograph imager, overcomes this problem by allowing the acquisition of whole-body radiograph images while the patient is in a weight-bearing position. It provides spinopelvic parameters and the degree of spondylolisthesis under physiological conditions similar to those of daily life.

To the best of our knowledge, no previous study used the EOS imaging system to assess the spinopelvic parameters in Eastern population. Thus, the aim of this study is to use the EOS imaging system to evaluate the spinopelvic parameters of spondylolysis patients, and their relationship with spondylolisthesis, disc degeneration, and age in a Taiwanese population.

## Materials and methods

Patients who visited our orthopedic clinic from December 2017 to June 2018 were included in this study. A total of 45 individuals complaining of low back pain and diagnosed with spondylolysis composed the spondylolytic group, while 32 asymptomatic individuals coming for conscription physical examination composed the control group. Plain radiography and computed tomography (CT) were used to confirm the diagnosis of spondylolysis. Imaging examinations undergone by the 77 patients, including radiography, EOS examination, and CT were clustered within an interval of one month. MRI performed in 33 of the 45 individuals with low back pain was analyzed as well. The images were examined and reported by board-certified musculoskeletal radiologists in NCKUH. Exclusion criteria for this study included patients with dysplastic, degenerative, or pathological spondylolisthesis, unilateral lysis, lumbosacral transitional vertebra, scoliosis, Scheuermann disease, hip pathology, and unidentifiable femoral heads on lateral radiographs. The gender, age, level of spondylolysis, degree of slip, degree of disc degeneration below the level of spondylolysis, and spinopelvic parameters were documented.

This research study was conducted retrospectively from data obtained for clinical purposes. We consulted extensively with the IRB of National Cheng Kung University Hospital who determined that our study did not need ethical approval or informed consent. An IRB official waiver of ethical approval was granted from the IRB of National Cheng Kung University Hospital. (No. B-ER-106-210)

### Radiographic Spinopelvic Parameters

Biplanar radiographs in a standing position were taken using the EOS imaging system (EOS Imaging). The subjects stood in a standardized erect posture, with knees and hips fully extended, shoulder anteriorly flexed by 90°, elbow extended, and hands resting on a support handle. Images were captured with minimal magnification and distortion as the system employs line detection of double-collimated X-ray beams. All radiographic parameters were measured by a senior orthopedist using the picture archiving and communication systems (PACS) software. The usefulness of these spinopelvic parameters were verified in previous studies by Tyrakowski et al. [16–18].

**Fig. 1:**
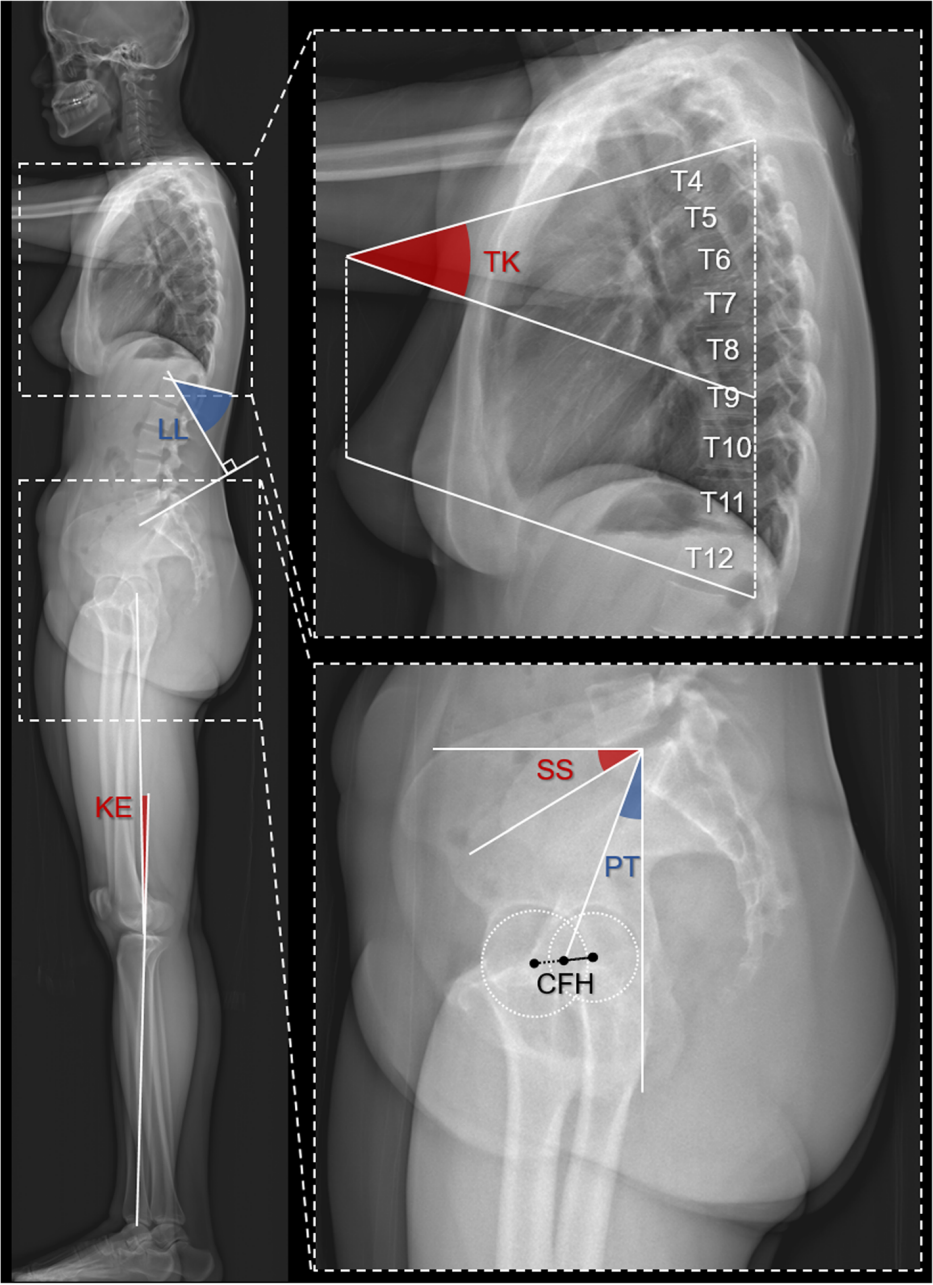
The graph shows how sagittal spinopelvic parameters were measured. PI = PT + SS. TK, thoracic kyphosis; PI, pelvic incidence; PT, pelvic tilt; SS, sacral slope; CFH, center of femoral heads; LL, lumbar lordosis; KE, knee extension

**Fig. 2:**
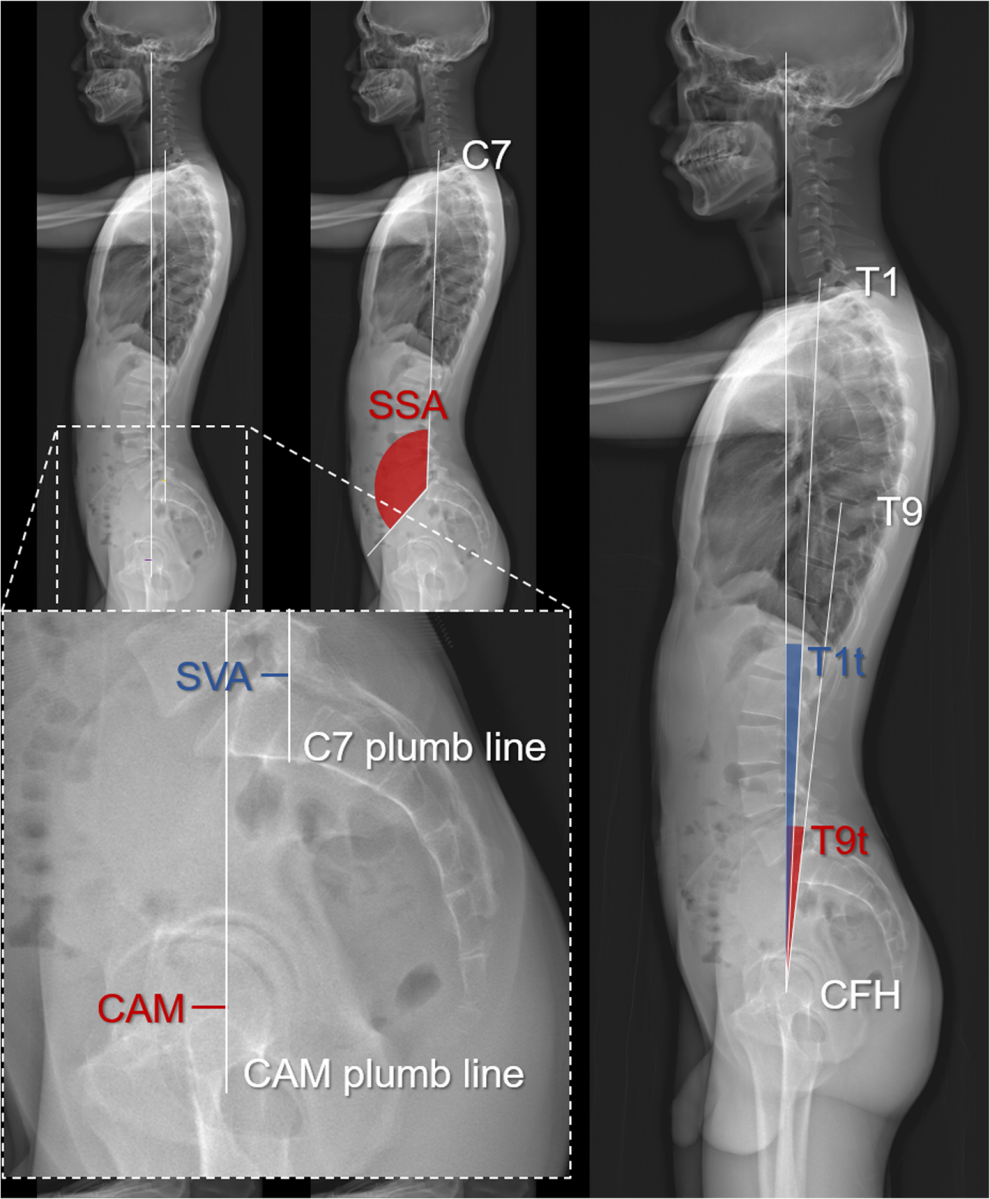
The graph shows how whole-body sagittal spinopelvic parameters were measured. SVA, sagittal vertical axis; CAM, center of acoustic meatus; SSA, spino-sacral angle; T1t, T1 tilt; T9t, T9 tilt; CFH, center of femoral heads

The following radiographic spinopelvic parameters were calculated (Figs. 1 and 2):

- Pelvic incidence (PI): the angle between the line perpendicular to the sacral endplate and the line connecting the midpoint of the femoral heads to the midpoint of sacral plate [9].
- Sacral slope (SS): the angle between the horizontal plane and the line tangent to the sacral endplate [9].
- Pelvic tilt (PT): the angle between the vertical line and the line connecting the midpoint of sacral endplate to the midpoint of the femoral heads, namely the anterior pelvic plane (APP) [9].
- Thoracic kyphosis (TK): the angle between the cranial T4 endplate and caudal T12 endplate [19, 20].
- Lumbar lordosis (LL): the angle between the cranial L1 endplate and the cranial sacral endplate [9].
- Sagittal vertical axis (SVA): the shortest distance between the posterosuperior corner of S1 and the C7 plumb line, which is the vertical line through the center of the C7 vertebral body [21]. (Fig. 2)
- Center of acoustic meatus (CAM) plumb line: the distance between the plumb line through CAM and the C7 plumb line.
- T1 tilt (T1t): the angle between the line tangent to the cranial endplate of T1 and a horizontal line.
- T9 tilt (T9t): the angle between the line tangent to the cranial endplate of T9 and a horizontal line.
- Spinosacral angle (SSA): the angle between the line connecting the center of the C7 vertebral body to the midpoint of the cranial sacral endplate and the line tangent to the cranial sacral endplate.
- Knee extension (KE): the angle between the axis of femur and that of the tibia (Fig. 1). The average of bilateral angles was used in statistical analysis.

### Analysis of Subgroups

The radiographic spinopelvic parameters of patients with spondylolysis and those of the control group were compared. The spondylolytic group was further divided according to the degree of spondylolisthesis, disc degeneration, and age.

First, the spondylolytic group was dichotomized according to the presence of spondylolisthesis. The spinopelvic parameters of the spondylolisthetic spondylolysis group and the simple spondylolysis group were analyzed.

Second, the IVD immediately inferior to the spondylolytic segment was assessed. Degeneration of the discs was graded according to the modified Pfirrmann classification on MRI images [22]. Patients with grade I, II, or III disc degeneration on the modified Pfirrmann classification were defined as having no high-grade degeneration, and those with grade IV or V disc degeneration were defined as having high-grade degeneration. The spinopelvic parameters for the low- and high-grade groups were compared.

Finally, the spondylolytic group was divided into a young group and a middle-aged group using an age of 30 years as the cutoff value. The division of age by 30 was based on a study conducted by Floman et al. in an Israeli population, who observed that spondylolisthesis tended to progress in the third decade of life [23].

### Statistical analysis

The spinopelvic parameters for the spondylolytic group and the control group were compared using the independent two-sample *t*-test. For subgroup analyses assessing the impact of spondylolisthesis, high-grade disc degeneration, and age, the Mann-Whitney U test was adopted. When comparing the grade of disc degeneration and the occurrence of spondylolisthesis between the young and middle-aged groups, Fisher’s exact test was used because the expected values in one of the cells were below 5. The level of significance was set at 0.05 (p < 0.05). All statistical analyses were performed using *SPSS* 17 (SPSS Inc., Chicago, Illinois).

## Results

The demographic data of the two groups are shown in Table 1. The mean age of the spondylosis group was higher than that of the control group.

**Table 1:** Demographics of the study population

		Spondylolysis group	Control group	
Number (n)		45	32	
Mean age (years)		34.9 ± 14.1	22.5 ± 4.2	
Male (n, %) Lysis level (n)		35 (78 %) L5 (40), L4 (5)	28 (87 %) 0	
Slip grade (n)		0 (16), I (27), II (2)	0	
IVD (n)		I (4), II (8), III (8), IV (11), V (2)	0	

## Analysis of spinopelvic parameters

Among the parameters, PI, LL, and KE were significantly higher in the spondylolysis group compared to those in the control group (Table 2). PI was 52.59 ± 11.6° in the spondylolysis group and 47.16 ± 7.95° in the control group (*p* < 0.05). LL was 41.51 ± 14.11° in the spondylolysis group and 28.22 ± 10.65° in the control group (*p* < 0.05). KE was 3.48 ± 2.14° in the spondylolysis group and 1.87 ± 4.48° in the control group (*p* < 0.01). In spondylolysis participants, there was no statistically significant difference in the measured spinopelvic parameters between slip and no slip (Table 3), nor between male and female subjects.

**Table 2:** Parameters for spondylolysis and control groups

		PI (°)	SS (°)	PT (°)	LL (°)	TK (°)	SVA (mm)	CAM (mm)	T1t (°)	T9t (°)	SSA (°)	KE (°)
Healthy control	Mean	47.16	37.91	9.47	28.22	34.50	9.94	-7.56	2.56	7.56	128.31	1.87
*n* = 32	SD	7.95	7.55	7.01	10.65	7.56	27.69	35.03	3.00	3.25	9.29	4.48
Spondylolysis	Mean	52.59	41.38	11.17	41.34	31.62	20.07	-5.48	2.04	6.48	130.33	3.48
*n* = 45	SD	11.60	8.74	6.66	14.11	10.98	25.77	23.46	2.14	3.62	9.53	2.14
*p* value		0.042*	0.123	0.279	0.040*	0.126	0.658	0.626	0.866	0.614	0.342	0.001*

*SVA* sagittal vertical axis; *CAM* center of acoustic meatus; *T1t* T1 tilt; *T9t* T9 tilt; *SSA* spinal-sacral angle; *KE* knee extension

* P < 0.05 was defined as statistically significant

**Table 3:** Parameters for spondylolysis patients with and without spondylolisthesis

		PI (°)	SS (°)	PT (°)	LL (°)	TK (°)	SVA (mm)	CAM (mm)	T1t (°)	T9t (°)	SSA (°)	KE (°)
Spondylolisthesis (-)	Mean	49.81	40.31	9.68	41.56	32.43	12.66	-8.91	2.41	7.16	130.75	3.58
*n* = 16	SD	11.57	10.05	5.57	14.29	7.15	12.98	17.85	1.31	2.08	11.61	2.64
Spondylolisthesis (+)	Mean	53.37	41.27	11.96	41.48	30.86	19.40	-9.40	2.36	7.00	129.45	3.50
*n* = 29	SD	11.62	8.10	7.15	12.69	14.27	30.17	29.08	2.53	4.16	7.99	2.46
*P* value		0.553	0.831	0.285	0.313	0.943	0.736	0.986	0.958	0.736	0.582	0.873

*SVA* sagittal vertical axis; *CAM* center of acoustic meatus; *T1t* T1 tilt; *T9t* T9 tilt; *SSA* spinal-sacral angle; *KE* knee extension

### Presence of high-grade intervertebral disc degeneration

Thirty-three spondylolysis participants received MRI examinations to assess the degree of disc degeneration. PI in those without high-grade IVD degeneration group was lower (50.54 ± 10.7°) compared to that in the high-grade group (58.38 ± 12.65°), though it did not reach a statistically significant difference (*p* = 0.087). There were no statistically significant differences between individuals with and without high-grade disc degeneration in all other parameters (Table 4). There was significantly more spondylolisthesis noted in high-grade IVD (92.3 %) compared to that in those without high-grade IVD (50 %) (*p* < 0.01).

**Table 4:** Parameters for high-grade and those without high-grade intervertebral disc degeneration in spondylolysis patients

Condition of IVD degeneration	Prevalence of Spondylolisthesis		PI (°)	SS (°)	PT (°)	LL (°)	TK (°)	SVA (mm)	CAM (mm)	T1t (°)	T9t (°)	SSA (°)	KE (°)
No High-grade	50 % (10/20)^a^	Mean	50.45	41.15	9.40	42.20	29.65	7.80	-16.80	2.93	6.80	131.60	3.06
*n* = 20		SD	10.70	8.75	5.68	13.04	8.84	17.05	16.40	1.66	3.14	9.46	2.40
High-grade	92.3 % (12/13)^b^	Mean	58.38	44.15	14.15	47.07	36.30	23.09	-9.18	2.54	8.27	131	3.45
*n* = 13		SD	12.65	8.07	8.02	16.38	14.42	30.65	32.04	2.38	3.16	7.57	2.38
*P* value	0.001^*^		0.087	0.334	0.110	0.501	0.221	0.164	0.610	0.507	0.413	0.760	0.507

*IVD* intervertebral disc; *SVA* sagittal vertical axis; *CAM* center of acoustic meatus; *T1t* T1 tilt; *T9t* T9 tilt; *SSA* spinal-sacral angle; *KE* knee extension

^*^P < 0.05 was defined as statistically significant

^a^ All of the 10 patients had grade 1 spondylolisthesis, according to the meyerding classification

^a^ Of the 12 patients, 10 had grade 1 and 2 had grade 2 spondylolisthesis

### Parameters for young and middle-aged participants

There were no statistically significant differences in spinopelvic parameters between these two groups. The occurrence of spondylolisthesis was significantly higher in the middle-aged group (80 %) than that in the young group (45 %) (*p* < 0.05) (Table 5). Among the 33 individuals (out of the 45 patients with low back pain) who underwent MRI examinations, a higher degree of IVD degeneration was found in the middle-aged group (52.4 %) compared with the young group (16.7 %) with a statistically significant difference (*p* < 0.05) (Table 6).

**Table 5:** Parameters for young and middle-aged participants

Age (years)	Prevalence of Spondylolisthesis		PI (°)	SS (°)	PT (°)	LL (°)	TK (°)	SVA (mm)	CAM (mm)	T1t (°)	T9t (°)	SSA (°)	KE (°)
**< 30**	45 %	Mean	50.40	39.75	10.60	39.85	30.40	13.26	-6.66	2.53	6.80	128.93	3.33
*n* = 20		SD	10.59	9.78	5.21	14.95	8.71	13.81	19.41	1.68	3.32	10.11	2.52
**> 30**	80 %	Mean	53.48	41.88	11.60	42.84	32.24	20.00	-11.2	2.26	7.26	130.68	3.68
*n* = 25		SD	12.39	7.89	7.70	13.57	12.62	31.86	29.64	2.51	3.76	8.75	2.51
*P* value	0.017^*^		0.664	0.598	0.748	0.458	0.900	0.732	0.560	0.681	0.758	0.758	0.632

*IVD*intervertebral disc; *SVA* sagittal vertical axis; *CAM* center of acoustic meatus; *T1t* T1 tilt; *T9t* T9 tilt; *SSA* spinal-acral angle; *KE* knee extension

^*^P < 0.05 was defined as statistically significant

**Table 6:** Disc degeneration in young and middle-aged participants

	No high-grade disc degeneration	High-grade disc degeneration^a^
Age < 30	10	2
Age > 30	10	11

*P* = 0.047 using Chi-Square analysis

^a^Grade I-III disc degeneration on the modified Pfirrmann classification were defined as having no high-grade degeneration, while grade IV-V were defined as having high-grade degeneration

## Discussion

Sagittal spinopelvic alignment has a profound impact on the biomechanics of the spine and environment and is of central importance in the pathogenesis of spondylolysis and spondylolisthesis. In the present study, PI, LL, and KE were significantly higher in spondylolytic individuals compared to healthy people. The patients with high-grade disc degeneration were more prone to spondylolisthesis (92.3 %) compared to those without (50 %). Spondylolytic patients older than 30 years old were also more prone to spondylolisthesis (80 %) and high-grade disc degeneration (52.4 %) compared to those younger than 30 years old (45 and 16.7 %, respectively). No significant difference in sagittal spinopelvic parameters was found when spondylolytic individuals were subdivided according to the presence of spondylolisthesis or high-grade disc degeneration.

It is well established that spinopelvic parameters of healthy individuals are, at least in part, associated with ethnic background. Zhu et al. compared healthy subjects from a Chinese Han population with age-matched controls from a Caucasian population. The PI and SS reported for the Chinese individuals, 44.6° and 32.5°, were significantly smaller than those for the Caucasian ones, 52.6° and 39.6°, respectively [22]. Interestingly, healthy young individuals from different Asian countries differ as well. A healthy control group from a Korean population had PI and SS of 45.8° and 29.3°, respectively [12], with the latter being somewhat smaller than that of a Chinese population. In the healthy control group of the current study, PI was 47.16° and SS was 37.91°, which are larger than those for other Asian individuals but smaller than those for Caucasian people according to statistics from previous studies [1, 14, 19, 24–28]. When making clinical decisions in a Taiwanese population, which has a diverse genetic background [29], extra caution should be taken if studies that focused on a non-Taiwanese ethnic group are referred to. To sum up, there are differences in spinopelvic alignment not only between Asian and Caucasian people but also among different ethnic groups in Asia.

The association between spinopelvic alignment and spondylolysis has been discussed in recent years. However, most studies considered only a Caucasian population, and the few studies that focused on an Asian population yielded inconsistent results. Among Caucasian patients, many studies found that PI and LL are larger among spondylolytic patients than among healthy individuals [1–3, 27], where both PT and SS were reportedly larger in spondylolysis patients in some of these studies [2, 27]. Yin et al. reported comparable results in a Chinese Han population, stating that PI, PT, SS, and LL of spondylolytic patients were all significantly higher than those of the healthy control group [9]. However, Oh et al. reported that only PI and SS were higher among Korean spondylolytic patients, while PT and LL did not differ significantly from those of healthy Koreans [12]. Among Taiwanese spondylolytic patients included in the current study, PI, LL, and SS were larger than those for the control group, though the last did not reach statistical significance. PT did not differ significantly from the control group. Our results are consistent with previous studies. Stagnara et al. proposed that LL increases linearly with SS and that the elevation in LL is to maintain overall balance [30–32]. The sameness of PT in our study could be partially accounted for by the theory of Oh et al., who illustrated that a compensatory mechanism such as PT did not occur until advanced age [12]. Notably, the control group showed slight mismatch of spinopelvic harmony, which was also found in a previous study on the Korean population [12]. It is possible that the range of spinopelvic harmony is slightly larger in the Asian population, but the speculation will require a larger population to confirm. Interestingly, the present study is the first to report that KE is increased in spondylolytic patients. Though the impact of KE angle on lower lumbar pathologies may not be substantial, the increase in KE may suggest one of the compensatory mechanisms after the incidence of spondylolysis. To conclude, the current study clarifies the association between spinopelvic alignment and spondylolysis in a Taiwanese population.

Parameters such as PI have been shown to increase in Caucasian patients with advanced isthmic spondylolisthesis [4, 6]. However, conflicting results have been observed in Asian populations. For patients with and without spondylolisthesis, Yin et al. did not report any difference in the sagittal lumbosacral parameters whereas Oh et al. identified an increase in PI, SS, and LL as spondylolisthesis progressed [9, 13]. In the present study, PI and LL were comparable in spondylolytic patients with and without slippage, which supports the distinction between Caucasian and Asian spinopelvic alignment. Interestingly, substantial but statistically insignificant increases in SVA were observed in spondylolytic patients, and further increases were found for patients with spondylolisthesis. Limited by the scope of conventional radiography, previous studies could not probe into the global indices of the body. The increase in SVA reflects that the center of gravity is shifted anteriorly in spondylolytic Taiwanese patients, and that this condition is exacerbated when spondylolisthesis occurs. In brief, Taiwanese patients with isthmic spondylolisthesis have comparable PI to that of the control group but their center of gravity drifts anteriorly.

The IVD is one of the major contributors to spine stability. When a vertebra slips anteriorly, the IVD is wedged and thus the LL increases to compensate for the shift of the center of gravity [28]. It is known that the disc immediately inferior to the spondylolytic level is prone to high-grade degeneration [10, 33–35]. Our results are consistent with this observation, demonstrating that high-grade disc degeneration is associated with spondylolisthesis while low-grade disc degeneration is not. This implies that without the stabilizing force from a healthy disc, the anterior sliding of isthmic spondylolisthesis could be intractable. Our study is consistent with the literature, confirming the major role of the IVD in spinal stability.

IVD degeneration is a complicated process, involving tissue damage and many age-related changes [36]. In addition, spondylolysis itself is a risk factor for disc degeneration, with approximately 15 % of spondylolytic patients eventually progressing to spondylolisthesis [37]. In the Taiwanese population in our study, patients in the middle-aged group were apt to have a higher degree of spondylolisthesis and a higher grade of disc degeneration. This finding is consistent with the natural course of spondylolysis; the percentage of spondylolisthesis and the grade of disc degeneration are expected to increase with age [38]. Hence, more attention should be paid to middle-aged Taiwanese spondylolytic patients. The follow-up interval should be shortened compared the length suggested in western literature, and back braces may be considered.

The EOS imaging system is one of the most pragmatic approaches for investigating spinopelvic parameters. Previous studies revealed that the EOS system can examine the whole body in a physiological stance and produce high-resolution images with little distortion while avoiding the high radiation dose associated with taking many conventional radiographs at once [15, 39]. These features allowed us to evaluate whole-body indices such as SVA. The EOS imaging system also provides high-quality lateral images of the pelvic girdle and lower extremities, which are difficult to obtain using conventional X-ray techniques [40]. The system thus allows clinicians to assess pelvic parameters in a reliable and reproducible manner. To the best of our knowledge, this is the first study to use the EOS imaging system for investigating radiographic parameters in individuals with spondylolysis. The present study demonstrated the consistency and reproducibility of EOS images.

There are several limitations of the current study. First, the cross-sectional rather than longitudinal design precluded the recognition of disease progression and the exact point in time of the occurrence of spondylolysis. Second, the sample size of our study may not be large enough. Though we observed that TK was larger in the spondylolytic group, we were unable to perform subgroup analyses on those with radicular symptoms because of the limited numbers. The observation need to be validated in a different, larger group. Third, we did not evaluate the symptom severity because back pain severity might not be proportional to image findings. Fourth, many of the subjects underwent EOS evaluation because of conscription physical examination, and were therefore male and young. Finally, MRI was only available in 33 out of the 45 patients with spondylolysis. The reduction in sample size may undermine the power of the current study.

## Conclusions

In a Taiwanese population, PI and LL were significantly larger in spondylolytic patients, which is different from reports for Caucasian populations. Disc degeneration and age were associated with the occurrence of spondylolisthesis. Ethnological differences should thus be taken into account when making clinical decisions regarding spondylolysis in a Taiwanese population.

## Data Availability

The datasets generated and analyzed during this study are available from the corresponding author on reasonable request.

## Abbreviations

PI: Pelvic incidence
SS: Sacral slope
PT: Pelvic tilt
TK: Thoracic kyphosis
LL: Lumbar lordosis
SVA: Sagittal vertical axis
CAM: Center of acoustic meatus
T1t: T1 tilt
T9t: T9 tilt
SS: A:spinosacral angle
KE: Knee extension
CR: Computed radiography
DR: Digital radiography
CT: Computed tomography
MRI: Magnetic resonance imaging
